# Knowledge of Male Infertility and Acceptance of Medical Assistance Reproductive Technology Among Fertile and Infertile Senegalese Men

**DOI:** 10.2147/RRU.S487854

**Published:** 2024-12-24

**Authors:** Oumar Gaye, Khadidiatou Ba, Mouhamed Diallo, Awa Niasse, Amdy Laye Counta, Modou Ndiaye, Moustapha Gning, Ablaye Gueye, Papa Ahmed Fall

**Affiliations:** 1 Urology Department of Dalal Jamm Hospital, Dakar, Senegal

**Keywords:** knowledge, male infertility, assisted reproductive technology, awareness, Senegal

## Abstract

**Purpose:**

The objective of our study was to examine the knowledge of male infertility and the acceptance of assisted reproductive technology (ART) methods.

**Patients and Methods:**

We conducted a descriptive, comparative, cross-sectional study from April 2023 to August 2023 in a center in Dakar. Included in the study were male patients aged 18 and older followed for male infertility (group 1) and fertile patients of the same age as those in group 1 (group 2). We designed a questionnaire assessing the sociodemographic characteristics of the respondents, their knowledge of male infertility, knowledge of ART, its acceptability, and the source of information about male infertility.

**Results:**

Our sample size consisted of 119 respondents for each group. The average age of respondents in both groups was 41.24 ± 8.42 years. Fifty-eight percent of respondents in group 1 were referred by their wife’s gynecologist. Fifty-four percent of respondents in group 1 had a good knowledge of male infertility, and 42.86% had average knowledge of male infertility. The majority of respondents in group 1 (42.9%) and group 2 (40.3%) did not know the duration that defines infertility. Fifty-seven percent of respondents in Group 1 and 81.5% of respondents in Group 2 did not know what assisted reproductive technology meant. Eighty-six percent of respondents in Group 1 agreed to use ART for procreation. The majority of respondents in Group 1 (54.6%) and Group 2 (58.8%) attributed a success rate of between 35% and 75% to ART.

**Conclusion:**

Infertile men had better knowledge of male fertility than fertile men. Respondents in both groups, as well as the advanced age of men did not know the duration defining infertility. Poor knowledge of ART was also observed among respondents in both groups, and a better acceptance of ART methods was noted among infertile men.

## Introduction

Infertility is defined as the inability to conceive a child after a year of regular, unprotected sexual intercourse. The prevalence of infertility is approximately 15% among couples,^1^ with a male factor identified in about 60% of cases. However, socio-cultural considerations of masculinity create psychosocial barriers to seeking professional health care.^2^ In Africa, women are often held responsible for infertility,^3^ despite best practice guidelines recommending that the initial assessment of a couple’s infertility be conducted simultaneously in both men and women.^4^ Moreover, infertility, recognized as a disease by the WHO,^5^ continues to be viewed within several African societies as the consequence of supernatural manifestations, such as evil spirits, witchcraft, or divine punishment.^6^

Lifestyle factors such as obesity, diet, smoking, excessive alcohol consumption, and environmental chemical exposures^7^,^8^ can impair male fertility. Thus, education on fertility issues is essential to avoid delays in seeking care and/or behaviors that impair fertility.^9^ This underpins the emphasis on lifestyle modifications in the new approaches to managing male infertility, thereby reducing the reliance on Assisted Reproductive Technology (ART).^10^ However, studies evaluating men’s knowledge of fertility are scarce, with most fertility knowledge studies conducted among women.

Despite the effectiveness of lifestyle changes in managing male infertility, ART remains a significant therapeutic means, enabling many couples to conceive.^11^ Nonetheless, access to and acceptability of ART are influenced by religious, cultural, political, and economic factors. The perspectives of different religions or cultures on ART vary greatly, affecting its acceptability. Additionally, its high cost, in a context of no social coverage or insurance, renders it inaccessible for most patients living in Africa.^12^

Given the importance of understanding male infertility factors for the preservation or restoration of male fertility, and since the acceptance of ART depends on factors such as religion or cost, it is necessary to evaluate these in Senegal, especially since this topic has been little studied in men in the literature.

Thus, our study aimed to assess the knowledge of male infertility and the acceptance of ART methods among fertile and infertile Senegalese men.

## Patients and Methods

### Type and Period of Study

This was a descriptive, comparative, and cross-sectional study aimed at evaluating the knowledge of male infertility and the acceptability of Assisted Reproductive Technologies (ART) among fertile and infertile patients followed at a university hospital in Dakar from April 2023 to August 2023.

### Population

**Figure 1:**
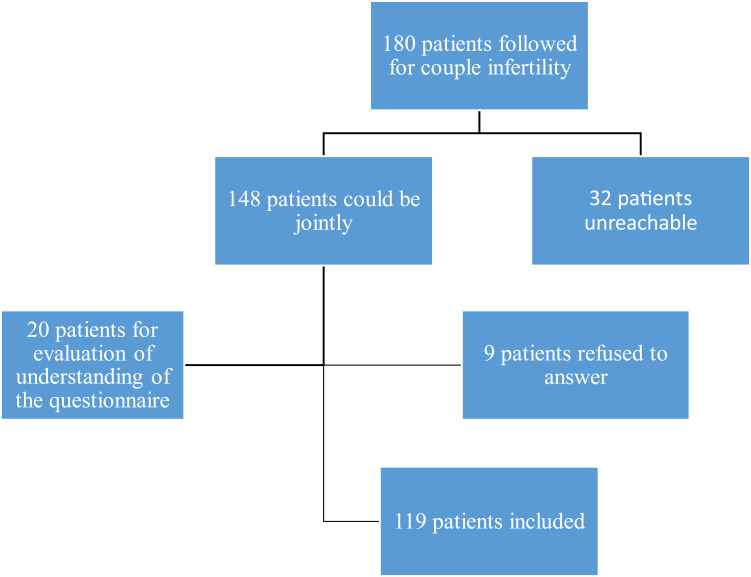
Distribution diagram of infertile respondents included in the study.

The included patients were those followed for male infertility, aged 18 years and older, at the urology service of a university hospital in Dakar from January 2019 to December 2022 (Group 1). The control group (Group 2) consisted of patients or companions of fertile patients without chronic pathology of the same age as the patients in Group 1, followed in outpatient consultation. Patients who did not have the faculties allowing them to understand and/or respond to the questionnaire were not included in the study. Among the 180 patients followed for male infertility in the urology-andrology service, 148 patients could be contacted. Among them, 139 patients had agreed to respond to the questionnaire, and the data from the first 20 respondents were used to evaluate the understanding of the questionnaire (Figure 1). In total, 119 infertile patients were included (Group 1) and compared to 119 control respondents of the same age (Group 2).

### Questionnaire

We designed a questionnaire based on previously published works on male infertility.^13–15^ This questionnaire was written in French and translated into Wolof (the national language of Senegal). The terms used were meticulously chosen according to the national dictionary to facilitate understanding of the questions. The questionnaire included 4 parts:

1. The first part of the questionnaire consisted of questions specifying the sociodemographic characteristics of the respondents (age of respondents, age of their wives, marital status, duration of marriage, type of infertility, educational level, religion, culture, occupation…).
2. The second part of the questionnaire consisted of questions assessing the knowledge of modifiable factors promoting male infertility (tobacco use, drug, and alcohol consumption, pathologies favoring male infertility such as diabetes, obesity, and genetic disease.)., the duration defining infertility, the period of female fertility, the perception of infertility as a disease (or due to a curse or a djinn). Responses were scored a 1 if the respondents had given the correct answer and 0 when an incorrect answer was given. For the 18 questions, a total score ranging from 0 to 18 could be assigned to each respondent. This total score allowed us to individualize 3 classes: poor knowledge (0 to 6), average knowledge (7 to 12), and good knowledge (13 to 18).
3. The third part of the questionnaire consisted of questions assessing the knowledge of ART (definition, cost, availability, accessibility, legal framework of ART with gamete donation in Senegal.), and its acceptability (with their gametes, with gamete donation, sperm or embryo conservation) or that of child adoption in case of failure or refusal of the latter.
4. The fourth part of the questionnaire specified the source of information of the respondents on male infertility (internet, doctor, family, friends).

### Definitions

#### Educational Level

Primary (Preschool—Elementary school), Secondary (College and high school), Tertiary (University or other post-secondary education)

#### Ethnicity

Senegal has multiple ethnic groups, the biggest ones being the Wolofs, the Fulanis, the Sereers and the Joolas.

#### Currency

The West African French franc (XOF) was the currency used to report the monthly salary of respondents.

#### Obedience

Religious brotherhoods or denominations in Senegal [Muslim (Tidianes, Mourides, Khadr…) and Christians (Catholics et Protestants)].

#### Djinns

Supernatural beings in African culture thought to be responsible for curses and misfortunes.

### Application of the Questionnaire to Respondents

Two investigators (Ms. K Ba and Mr. A Counta) were trained to reduce the bias of understanding the questions by the respondents. The two investigators applied the questionnaire to each participant interviewed for about 20 minutes.

### Data Collection

Data from the questionnaire applied to respondents were collected on an online platform named “Kobotoolbox.”

### Statistical Analyses

The software SPSS version 2.0 was used for analyses. Associations were evaluated using the chi-squared test. A p-value of ≤ 0.05 was considered statistically significant.

### Ethical Considerations

Informed consent was obtained from all participants before the interview. Participants had the right to withdraw from the study without explaining to the research team. Confidentiality of the obtained data was guaranteed.

## Results

### Sociodemographic Characteristics of Respondents (Table 1)

The average age of respondents in both groups was 41.24 ± 8.42 years (ranging from 24 to 61 years). Age groups between 30 to 40 years and 40 to 50 years were predominant, respectively, in 37% and 37.8% of cases. The average age of the wives of Group 1 respondents was 31.79 ± 7.5 (ranging from 18 to 55 years), and for Group 2 respondents, it was 32.82 ± 7.52 (ranging from 20 to 55 years). Eighty-four percent of Group 1 respondents and 85.7% of Group 2 respondents were married under monogamous arrangements. Infertility was primary in 79% of the cases for Group 1 respondents. Their wife’s gynecologist referred Fifty-eight percent of Group 1 respondents.

**Table 1:** Comparison of Sociodemographic Characteristics of the Two Groups

	Group		P
	Group 1 n (%)	Group 2 n (%)	
**Age of respondents (years)**			1
<30	9 (7.6)	9 (7.6)	
[30–40]	44 (37.0)	44 (37.0)	
[40–50]	45 (37.8)	45 (37.8)	
[50–60]	19 (16.0)	19 (16.0)	
>60	2 (1.7)	2 (1.7)	
**Age of spouses (years)**			0.489
< 20	3 (2.3)	0 (0.0)	
[20–30]	50 (37.6)	41 (34.5)	
[30–40]	56 (42.1)	55 (46.2)	
[40–50]	22 (16.5)	20 (16.8)	
>50	2 (1.5)	3 (2.5)	
**Type of marriage**			0.855
Monogamous	101 (84.9)	102 (85.7)	
Polygamous	18 (15.1)	17 (14.3)	
**Duration of marriage (years)**			0.041
< 5	35 (29.4)	23 (19.3)	
[5–10]	49 (41.8)	47 (39.5)	
> 10	35 (29.4)	49 (41.2)	
**Type of infertility**			NA
Primary infertility	94 (79.0)	NA	
Secondary infertility	25 (21.0)	NA	
**Education level**			0.22
Primary	17 (14.3)	19 (16.0)	
Secondary	81 (68.1)	69 (58.0)	
Tertiary	21 (17.6)	31 (26.0)	
**Religion**			0.115
**Muslim**	110 (92.4)	115 (96.6)	
Khadre	5 (4.5)	13 (11.3)	
Mouride	46 (41.8)	49 (42.6)	
Tidiane	53 (48.2)	40 (34.8)	
Others	6 (5.5)	13 (11.3)	
**Christian**	9 (7.6)	4 (4.4)	
Catholics	9 (100)	3 (75.0)	
Protestants	0 (0.0)	1 (25.0)	
**Ethnicity**			0.036*
Wolof	46 (38.7)	36 (30.3)	
Peul	26 (21.8)	25 (21.0)	
Sérère	14 (11.8)	9 (7.6)	
Diolas	5 (4.2)	1 (0.8)	
Autres	28 (23.5)	48 (40.3)	
**Occupation**			0.025*
Unemployed	6 (5.0)	16 (13.4)	
Employed	113 (95.0)	103 (86.6)	
**Monthly income (FCFA)**			0.58
<100,000	50 (42.0)	42 (35.3)	
100,000–300,000	14 (11.8)	14 (11.8)	
300,000–500,000	52 (41.7)	48 (40.3)	
>500,000	3 (2.5)	15 (12.5)	
**Health insurance**			0.06
No	99 (83.2)	87 (73.1)	
Yes	20 (16.8)	32 (26.9)	
**Reason for consultation**			
Referred by wife’s gynecologist	69 (58)		
Spontaneously	50 (42)		
Other	0 (0)		

**Notes**: P-value found using a Chi2 test. *Significant statistical difference was found.

### Assessment of Male Infertility Knowledge (Table 2)

**Figure 2:**
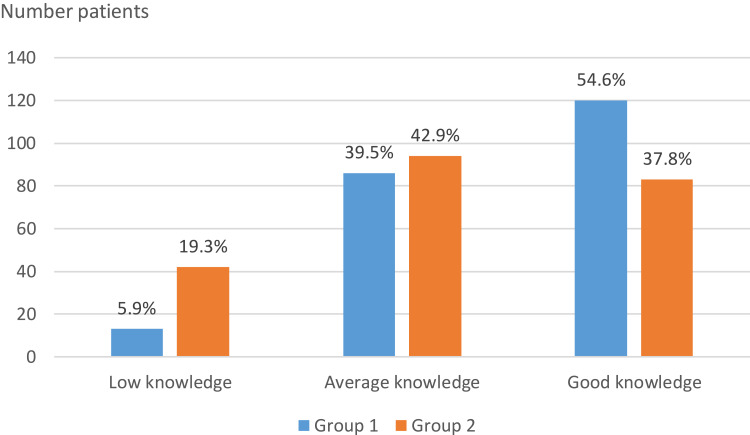
Distribution of respondents in groups 1 and 2 according to their knowledge of infertility.

Group 1 respondents had an average total knowledge score of male infertility of 12.49 ± 3.42, and Group 2 respondents had an average total score of 10.97 ± 4.41. Fifty-four percent of Group 1 respondents had good knowledge of male infertility (total score between 13 and 18), and 42.86% of Group 1 respondents had average knowledge of male infertility (total score between 7 and 12) (Figure 2). Unlike Group 1 respondents, the majority of Group 2 respondents did not know that tobacco, drug use, and excessive alcohol consumption could impair male fertility. The majority of respondents from both groups knew that the use of anabolic steroids, exposure to certain toxic products, or taking certain medications could compromise male fertility. Most respondents from both groups did not recognize advanced age as a factor favoring male infertility. The majority of Group 1 respondents (42.9%) and Group 2 (40.3%) did not know that infertility is discussed after 12 months of unprotected sexual intercourse without conceiving.

**Table 2:** Distribution of Respondents in the Two Groups According to Their Knowledge of Male Fertility

	Group 1 n(%)	Group 2 n(%)	P
**Smoking**			<0.001*
Yes	84 (70.6)	45 (37.8)	
No	35 (29.4)	74 (62.2)	
**Drug use**			<0.001*
Yes	83 (69.7)	46 (38.7)	
No	36 (30.3)	73 (61.3)	
**Excessive alcohol consumption**			<0.001*
Yes	82 (68.9)	52 (43.7)	
No	37 (31.1)	67 (56.3)	
**Advanced age of the man**			0.141
Yes	39 (32.8)	50 (42.0)	
No	80 (67.2)	69 (58.0)	
**Genital infection**			<0.001*
Yes	100 (84.0)	66 (55.5)	
No	19 (16.0)	53 (44.5)	
**Genital malformation**			0.122
Yes	103 (86.6)	94 (79.0)	
No	16 (13.4)	25 (21.0)	
**Obesity**			0.76
Yes	92 (77.3)	90 (75.6)	
No	27 (22.7)	29 (24.4)	
**Genetic disease**			0.275
Yes	96 (80.7)	89 (74.8)	
No	23 (19.3)	30 (25.2)	
**Heat exposure (job exposing to heat, tight underwear)**			0.075
Yes	85 (71.4)	72 (60.5)	
No	34 (28.6)	47 (39.5)	
**Anabolic steroid use**			0.458
Yes	91 (76.5)	86 (72.3)	
No	28 (23.5)	33 (27.7)	
**Exposure to certain toxic products**			0.07
Yes	88 (73.9)	75 (63.0)	
No	31 (26.1)	44 (37.0)	
**Duration defining infertility**			0.429
Yes	46 (38.7)	52 (43.7)	
No	73 (61.3)	67 (56.3)	
**Exposure to certain toxic products**			0.685
Yes	78 (65.5)	75 (63.0)	
No	41 (34.5)	44 (37.0)	
**Diabete**			0.341
Oui	81 (68.1)	74 (62.2)	
Non	38 (31.9)	45 (37.8)	
**Period of fertility**			0.367
2–3 days every month	85 (71.43)	76 (63.7)	
15 days every month	31 (26.05)	42 (35.29)	
All of the month	3 (2.52)	1 (0.84)	
**Duration defining infertility**			0.22
24 months	27 (22.69)	40 (33.61)	
18 months	22 (18.49)	19 (15.97)	
12 months	51 (42.86)	48 (40.34)	
6 months	19 (16.97)	12 (10.08)	
**Infertility is**			0.219
Due to djinns	1 (0.84)	0 (0.0)	
A disease	116 (97.48)	119 (100.0)	
Due to a curse	2 (1.68)	0 (0.0)	
**Infertility is curable**			0.122
Yes	87 (73.1)	97 (81.5)	
Not sure	32 (26.9)	22 (18.5)	
No	0 (0)	0 (0)	

**Notes**: P-value found using a Chi2 test. *Significant statistical difference was found.

### Assessment of Assisted Reproductive Technologies (ART) Knowledge

Fifty-seven percent of Group 1 respondents and 81.5% of Group 2 respondents did not know what Assisted Reproductive Technology (ART) meant (Table 3). After explaining ART and its various methods, 65% of Group 2 respondents did not consider ART as a natural method (Table 3). The majority of Group 1 (54.6%) and Group 2 (58.8%) respondents attributed an ART success rate between]35–75%].

**Table 3:** Distribution of Respondents in the Two Groups According to Their Acceptance of Assisted Reproductive Technology (ART) Methods

	Group 1 n(%)	Group 2 n(%)	P
**Knowledge of ART**			
Yes	51 (42.9)	22 (18.5)	< 0.001*
No	68 (57.1)	97 (81.5)	
**Do you think that ART with egg/sperm donation is legal in Senegal?**			
Yes	17 (14.3)	5 (4.2)	
No	41 (34.5)	68 (57.1)	< 0.001*
Uncertain	61 (51.3)	46 (38.7)	
**Do you think that ART is a natural means?**			
Yes	61 (51.3)	12 (10.1)	
No	21 (17.6)	78 (65.5)	< 0.001*
Uncertain	37 (31.1)	29 (24.4)	
**Is ART disponible in Sénégal ?**			
Yes	47 (39.5)	24 (20.2)	< 0.001*
No	9 (7.6)	26 (21.8)	
Uncertain	63 (52.9)	69 (58.0)	
**Do you think that ART is accessible to those who want it?**			
Yes	54 (45.4)	17 (14.3)	< 0.001*
No	5 (4.2)	24 (20.2)	
Uncertain	60 (50.4)	78 (65.5)	
**Does the advanced age of the man influence the success rate of ART?**			
Yes	27 (22.7)	15 (12.6)	0.047*
No	71 (59.7)	71 (59.7)	
Uncertain	21 (17.6)	33 (27.7)	
**What do you think of the cost of ART?**			
Cost	58(48.7)	45(37.8)	0.07
Uncertain	59 (49.6)	74(62.2)	
Not cost	2(1.7)	0(0.0)	
**Would you agree to carry out ART there?**			
Yes	103(86.6)	1(0.8)	<0.001*
No	16(13.4)	118(99.2)	
**Would you do ART with sperm donation?**			
Yes	24(20.2)	0(0.0)	<0.001*
No	81(68.1)	101(84.9)	
Uncertain	14(11.8)	18(15.1)	
**Would you do ART with egg donation?**			
Yes	24(20.2)	1(0.8)	<0.001*
No	81(68.1)	100(84.0)	
Uncertain	14(11.8)	18(15.1)	
**Would you do ART with your own sperm and your wife’s egg?**			
Yes	97(81.5)	1(0.8)	
No	19(16.0)	99(83.2)	<0.001*
Uncertain	3(2.5)	19(16.0)	
**Would you preserve your sperm for possible future use?**			
Yes	61(51.3)	1(0.8)	
No	45(37.8)	101(84.9)	<0.001*
Uncertain	13(10.9)	17(14.3)	
**In case of multiple fertilization during ART, would you keep the eggs (embryos)?**			
Yes	84(70.6)	1(0.8)	<0.001*
No	21(17.6)	111(93.3)	
Uncertain	14(11.8)	7(5.9)	

**Notes**: P-value found using a Chi2 test. *Significant statistical difference was found.

### Assessment of Acceptance of ART Methods (Table 3)

Eighty-six percent of Group 1 respondents were willing to use ART methods for conception. Conversely, only 0.8% of Group 2 respondents agreed to use them. Twenty percent of Group 1 respondents were agreeable to undergoing ART with gamete donation. Fifty-one percent and 70.6% of Group 1 respondents were respectively agreeable to sperm conservation and embryo conservation in cases of multiple fertilized egg formation.

### Assessment of Child Adoption Acceptance

**Figure 3:**
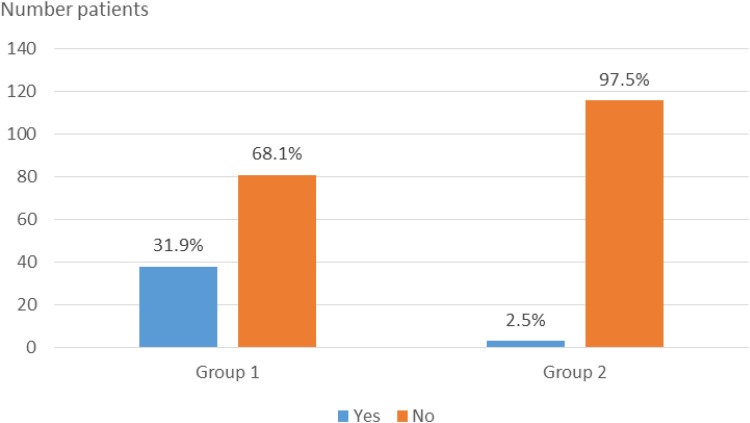
Distribution of Group 1 and 2 respondents according to their acceptance of adoption of children.

Only 31.9% of Group 1 respondents and 25% of Group 2 respondents would agree to adopt a child as an alternative (Figure 3).

### Factors Favoring Knowledge of Male Infertility, ART, and Its Acceptance

The educational level of respondents from Group 1 influenced their level of knowledge of ART (p=0.018) (Table 4). The educational level of respondents from Group 2 affected their knowledge of both infertility and ART (p=0.002 and p<0.001) (Table 5). However, religion, culture, or marriage duration did not influence the acceptance of ART and child adoption among respondents from both groups.

**Table 4:** Factors Influencing Knowledge of Infertility and Acceptance of ART Methods Among Group 1 Respondents

	Religion	Obedience	Ethnicity	Educational level	Patient age	Duration of marriage
Knowledge of infertility	0.63	0.793	0.841	0.175	0.018*	0.861
ART knowledge	0.169	0.044*	0.519	0.001*	0.088	0.331
ART acceptance	0.347	0.255	0.854	0.391	0.452	0.24
Acceptance of sperm donation	0.522	0.011	0.669	0.242	0.667	0.556
Acceptance of egg donation	0.552	0.302	0.669	0.242	0.667	0.556
Adoption acceptance	0.311	0.71	0.128	0.725	0.462	0.778

**Notes**: P-value found using a Chi2 test. *Significant statistical difference was found. Obedience: Religious brotherhoods or denominations in Senegal [Muslim (Tidianes, Mourides, Khadr…) and Christians (Catholics et Protestants)].

**Table 5:** Factors Influencing Knowledge of Infertility and Acceptance of ART Methods Among Group 2 Respondents

	Religion	Obedience	Ethnicity	Educational level	Patient age	Duration of marriage
Knowledge of infertility	0.603	0.219	0.34	0.002*	0.353	0.561
ART knowledge	0.02*	0.314	0.979	0.001*	0.348	0.71
ART acceptance	0.966	0.595	0.015*	0.694	0.751	0.462
Acceptance of sperm donation	NA	NA	NA	NA	NA	NA
Acceptance of egg donation	NA	NA	NA	NA	NA	NA
Adoption acceptance	0.098	0.282	0.417	0.328	0.757	0.757

**Notes**: P-value found using a Chi2 test. *Significant statistical difference was found Obedience: Religious brotherhoods or denominations in Senegal [Muslim (Tidianes, Mourides, Khadr…) and Christians (Catholics et Protestants)].

### Evaluation of the Source of Information on Male Infertility Among Respondents

**Figure 4:**
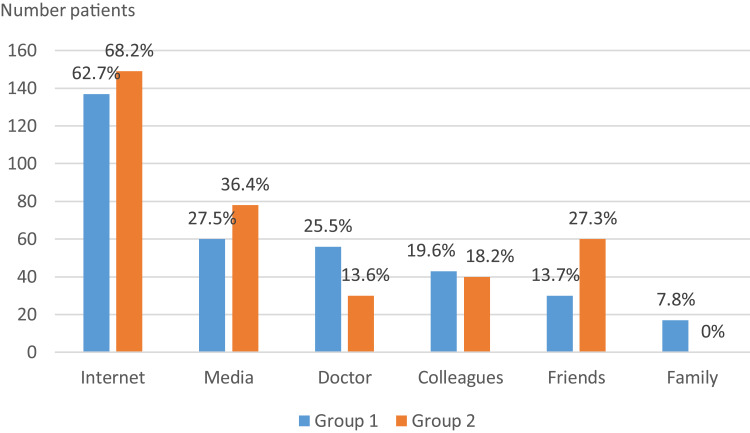
Distribution of respondents in groups 1 and 2 according to their source of information regarding male infertility.

The internet was the most reported source of information on male infertility and ART by 62.7% and 68.2% of respondents from Groups 1 and 2, respectively (Figure 4).

## Discussion

We interviewed 119 infertile men and 119 fertile men of the same age to compare their knowledge of male infertility and Assisted Reproductive Technology (ART). Few studies have focused on men’s knowledge of their fertility. We found that infertile men had better knowledge of male fertility than fertile men did. Despite a relatively good understanding of most modifiable factors of male infertility by respondents from both groups, the duration defining infertility and advanced male age as a factor favoring male infertility were not known to the respondents. Additionally, a poor understanding of ART was observed among respondents from both groups, with a better acceptance of ART methods among infertile men (Group 1).

Their wife’s gynecologist referred 58% of respondents from Group 1. Indeed, when a couple faces infertility, it is often the woman who is blamed, and men feel less concerned by infertility.^16^ The partner’s gynecologist asks the man to perform a semen analysis and refers him to andrology in case of abnormalities. We might be tempted to believe that this is a phenomenon observed only in Africa, but, unfortunately, the same observation has also been made in developed countries with respondents having a supposedly higher level of community health. This is due to male infertility affecting a man’s virility, giving him a sense of reduced masculinity, leading to a complex and difficulty in seeking medical help.^17^

Monogamy was the most observed type of marriage among respondents from both groups. However, one might have been tempted to think that polygamy would be more observed among respondents from Group 1 than those from Group 2 because one of the attitudes of men in the event of couple infertility in Africa is to seek another wife or to divorce.^18^

Respondents from Group 1 had a better knowledge of infertility factors than those from Group 2. This is probably because patients from Group 1 informed themselves more about infertility and had the opportunity to meet a doctor, as evidenced by the question about their source of information concerning infertility.

Only about 40% of respondents knew that couple infertility is discussed after 12 months of regular, unprotected sexual intercourse. This lack of knowledge contributes to a delay in consultation, as evidenced by numerous African series where this delay often exceeds 5 years.^19^ This delay in consultation reduces the chances of conceiving because the older a man gets, the more his chances of procreating are reduced.^20^

Unlike respondents from Group 1, respondents from Group 2 did not know that tobacco, drug use, and excessive alcohol consumption affect male fertility. It is important for men to know these modifiable factors of infertility because stopping these substances often leads to an improvement in the number and quality of spermatozoa. Respondents in both groups correctly identified psychoactive drug consumption, diabetes, obesity and use of anabolic steroids as factors affecting male fertility.

As in many studies, the majority of respondents from both groups did not recognize advanced male age as a factor favoring infertility.^21^ However, the quantity and quality of sperm decrease with age, and men should be sensitized to this. Indeed, men, like women, tend to form couples later in life nowadays, wishing to acquire certain financial stability before committing to a relationship and/or and having children.

Two respondents from Group 1 claimed that infertility was due to a curse, and one respondent attributed it to djinns. This perception of infertility as a curse or a spell cast on them has been observed in respondents from several African series.^6^,^19^ This perception often leads men to first seek or concurrently use traditional medicine.^22^ Respondents from other series believed that infertility was due to divine will and that over time and with prayers, their dream of procreating could become a reality if God so decided. This attitude also delays seeking care from a healthcare provider.

Like in our study, respondents from Canadian studies knew most of the modifiable factors of male infertility such as smoking, excessive alcohol consumption, or sexually transmitted infections.^14^,^23^ Most respondents were uncertain about the availability, accessibility, and cost of ART in Senegal. This could be explained by the fact that there was only one center performing ART in Senegal, and it was private, even though a public center would soon open in a hospital in Dakar. Currently, the cost of ART per oocyte retrieval is about 2,500,000 CFA francs, and it is not covered by Senegalese insurance. This economic aspect should be considered in setting the rates in this new center when it opens. Indeed, one of the major problems of ART in southern countries is the high cost.^24^ A study showed that the direct medical costs paid by patients living in low and middle-income countries for ART are often higher than the Gross Domestic Product (GDP) per capita, making it inaccessible to most patients.^12^

Moreover, we noticed that respondents from both groups overestimated the success rate of ART by oocyte retrieval. This observation has been made in several studies conducted in different countries.^13^ However, the chances of success per oocyte retrieval are about 25 to 30%.^25^

Eighty-six percent of respondents from Group 1 were willing to undergo ART compared to 8% of respondents from Group 2. This significant difference between the respondents of the two groups could be explained by a lesser knowledge of ART by respondents from Group 2 but also by the fact that they were not infertile and thus did not appreciate the opportunity that ART could offer them for procreation.

Nonetheless, no relationship between religion and the acceptance of ART was observed. This could be explained by an uneven distribution of our population, predominantly Muslim. However, the views of the Catholic Church and Islam differ on this issue. Indeed, the Vatican categorically opposes any ART technique because it believes that the dignity of the embryo must be respected as a person and that procreation must take place within marriage and in conjugal love presented as a reciprocal self-giving key to the relationship between spouses and to the relationship with God.^26^ Islam permits Assisted Reproductive Technology (ART) exclusively for married couples.

Despite a significant rate of acceptance of ART among respondents from Group 1, only 20.2% of respondents were open to undergoing ART with gamete donation. Our findings contrast with a study conducted in Nigeria, where 59% of respondents^27^ were favorable to gamete donation.

The alternative of child adoption in the event of refusal or failure of ART was only considered by respectively 31.9% and 25% of respondents from groups 1 and 2. These results are similar to the studies by Ezugwu^28^ conducted in Nigeria and by Bokaie in Iran,^29^ where respectively 59% and 82% of respondents were hesitant to adopt a child.

The internet was the primary source of information for respondents from both groups regarding their knowledge of male infertility and its management. Indeed, the internet has significantly changed how individuals obtain information about their health, and male infertility is no exception, as infertile men increasingly turn to social media for information, advice, or to share experiences.^13^,^30^ However, some information available on the internet may be inaccurate or misleading, as confirmed by studies evaluating the quality of information on male infertility available online.^13^ This contributes to the misinformation of patients with significant repercussions, such as in advertisements that overestimate the results of ART or dietary supplements that improve sperm quality. Therefore, it’s crucial for practitioners managing male infertility to communicate more through the internet, as it has become the preferred source of information for patients.

The sample was not representative of the general Senegalese population therefore these results cannot be generalized to the entire country. However the present study brings important information on a topic that has not been studied much in Subsaharan Africa. Methodologically, we chose to perform semi-structured interviews to inquire about our patients’ knowledge on male infertility and their acceptance of MAR. This format can stop respondents from fully expressing their perceptions during the interview particularly regarding current marital status and their perception of infertility as a disease.Although minimized by the training sessions and a structured interview, interviewer bias can still be present.

## Conclusion

Infertile men had a better understanding of male fertility than fertile men. Despite a relatively good knowledge of most modifiable factors of male infertility by respondents from both groups, the duration defining infertility, the respondents did not know advanced male age, and consanguinity as factors favoring male infertility. Additionally, a poor understanding of ART was observed among respondents from both groups, with a better acceptance of ART methods among infertile men. Awareness of male fertility among Senegalese men and its management options, such as lifestyle modifications or ART, needs to be raised. However, this awareness should be achieved using internet, as it is the primary source of information on infertility for Senegalese men.
